# Portuguese version of the Expanded Prostate Cancer Index Composite for Clinical Practice (EPIC-CP): psychometric validation and prospective application for early functional outcomes at a single institution

**DOI:** 10.1186/s12894-020-00734-y

**Published:** 2020-10-20

**Authors:** Danilo B. Lourenço, Breno Santos Amaral, Wladimir Alfer-Junior, Ana Vasconcellos, Fernanda Russo, Rafael Sanchez-Salas, Bianca Bianco, Andrew A. Wagner, Peter Chang, Marcio Covas Moschovas, Gustavo Caserta Lemos, Arie Carneiro

**Affiliations:** 1 Hospital Israelita Albert Einstein, Av. Albert Einstein, 627, Sala 303, Bloco A1, São Paulo, SP Brazil; 2 Institut Mutualiste Montsouris, Paris, France; 3 Beth Israel Medical Center, Harvard Medical School, Boston, MA USA

**Keywords:** EPIC, Prostate cancer, Quality of life

## Abstract

**Background:**

The Expanded Prostate Index Composite for Clinical Practice (EPIC-CP) is a short version of the original EPIC, developed to facilitate the instrument’s use in routine care. This study aimed to validate the EPIC-CP Portuguese version, and evaluate its role in presenting early functional outcomes of surgically treated prostate cancer patients at a Latin American referral center.

**Methods:**

The EPIC-CP was self-administered prospectively and individually by all localized prostate cancer patients, before and after robotic-assisted radical prostatectomy, from March 2017 to June 2018 at a single institution. For validation, we used the Cronbach’s alpha coefficient to evaluate internal consistency. The EPIC-CP domains were compared before surgery, and 6 months and 12 months after surgery. Statistical analyses were performed using the student’s *t* test, and Wilcoxon and Friedman tests, with *p* values < 0.05 considered significant.

**Results:**

One hundred and fifty two patients answered the EPIC-CP. The patients had a median age of 62.7 (± 8.5) years and prostate specific antigen level of 6.3 (± 4.6) ng/ml. The Cronbach’s alpha varied from 0.75 to 0.77 for all domains with good internal consistency, except for the “vitality/hormonal” domain, which had a score of 0.35. The domain evolution for the preoperative and 6-month postoperative groups revealed that the domains related to urinary continence and bowel worsened, and were increased during the first 6 months; however, this variation had no obvious clinical implications, and the irritative symptoms improved. Regarding the sexual domain, the scores worsened, and also increased over the first 6 months. The results of the confirmatory factor analysis were robust, with an explained variance of 0.951 and covariance of 0.929.

**Conclusions:**

The Portuguese version of the EPIC-CP is a reliable and valid questionnaire for postoperative patients, and very useful to improve the knowledge of the early functional outcomes of men treated for prostate cancer.

## Background

Patients diagnosed with prostate cancer have a high cancer-specific survival rate, and this combined with early detection could mean that many patients endure the treatment consequences for many years [1]. Currently, a large variety of treatments is available; hence, patient expectations and quality of life (QoL) have become essential tools that guide the decision-making of the medical staff regarding clinical management and treatment [2].

It is essential to use validated questionnaires to measure and objectively quantify the QoL. However, because the currently available questionnaires are long and require considerable time to complete, most are used only in clinical research [3].

The widely used generic questionnaires SF-36 (Medical Outcomes Study 36-Item Short-Form Health Survey) [4] and SF-12 (12-Item Short-Form Health Survey) [5] evaluate different dimensions of influence on quality of life by considering an individual's perception of health aspects over the previous 4 weeks. The lack of an instrument with an international perspective led the World Health Organization (WHO) to form a Quality of Life Group. This group developed a questionnaire composed of 100 questions to assess general quality of life, called the World Health Organization Quality of Life (WHOQOL) [6]. Several instruments were developed to measure health-related quality of life. The need for short instruments led the WHOQOL Group to develop an abbreviated version of the WHOQOL-100, the WHOQOL-bref [7].

In 1998, Litwin et al. [8] developed an instrument to capture the health concerns central to the quality of life of men treated for early stage prostate cancer, known as the University of California-Los Angeles Prostate Cancer Index (UCLA-PCI). The UCLA-PCI comprises six scales containing 20 disease-targeted items that address impairment in the urinary, bowel, and sexual domains. However, existing health-related quality of life instruments for prostate cancer patients failed in the assessment of irritative and obstructive urinary symptoms (complementing concurrent incontinence assessment), function-related issues, and specifically, hormone therapy effects and their related issues. To address these limitations, a broad-based modification of the UCLA-PCI was performed to derive the Expanded Prostate Cancer Composite Index (EPIC) [9].

EPIC comprises of 50 questions evaluating the five clinical domains of urinary incontinence, obstructive urinary symptoms, intestinal symptoms, sexual symptoms, and hormonal symptoms, each of which is given a specific score [9]. This questionnaire has been validated in different languages, including Portuguese [10], and is widely used in clinical research. However, because it includes a large number of questions that take a long time to complete, its use in daily clinical practice is not feasible.

A summarized version of the EPIC was formulated to improve and facilitate its application. The EPIC-26 is a validated and abbreviated form of the EPIC-50, comprising of 26 questions extracted from the original EPIC [11]. This new instrument includes the same five domains but with different items: urinary incontinence (4 items), urinary irritation/obstruction (4 items), intestinal symptoms (6 items), sexual function (6 items), and vitality/hormonal function (5 items).

This simplified version of the EPIC requires approximately 10 min to complete, and can be answered by phone or computer, making it easy to use in research. However, its use in clinical practice remains challenging due to the average response time required and the number of questions it contains [3]. To obtain a quick and easy questionnaire for use in research and clinical practice, the authors of the EPIC-26 developed the EPIC for Clinical Practice (EPIC-CP) (Additional file 2) [12].

The EPIC-CP questionnaire is self-administered, and it is intended to evaluate the impact of treatment on the QoL of prostate cancer patients. It includes 16 questions derived from the original EPIC and EPIC-26. These questions are divided into four domain symptoms: urinary, intestinal, sexual, and hormonal. The questionnaire evaluates the patient’s experiences in the last 4 weeks, and includes a Likert response scale with five options. The “urinary” domain has two additional sub-scales: incontinence and obstructive symptoms [12].

The EPIC-CP validation study [1] indicated that 77% of the patients completed the form in less than 5 min, the questionnaire is a sensitive and practical tool that can be efficiently administered in outpatients, and it allows results to be easily measured and documented during implementation. Therefore, the EPIC-CP provides an opportunity to incorporate health-related QoL in the clinical care of prostate cancer patients, and facilitates the implementation and documentation of patient data.

The main objective of this study was to validate the EPIC-CP in the Portuguese language, and to demonstrate early functional results in prospective analysis at a single Latin American institution.

## Methods

We conducted a prospective study in partnership with the EPIC and EPIC-CP authors and creators, who not only discussed the study design and methodology, but also authorized the validation of the Portuguese version.

In this study, from March 2017 to June 2018, 402 patients with prostate cancer from the Urology and Oncology clinic of the Hospital Israelita Albert Einstein, São Paulo, Brazil, were enrolled to take part of this study, which was approved by the hospital Ethics Committee (Approval number: 70687817.2.1001.0071). The questionnaires were self-administered and assigned to patients who agreed to the informed consent terms.

The questionnaire was administered to 402 preoperatively, and then to 152 of these patients 6 months after robotic radical prostatectomy, and 35 of them 12 months after undergoing that surgery. The Israelita Albert Einstein Hospital has a nationwide robotic surgery reference training center. Consequently, several uro-oncology services throughout Brazil refer their patients for surgical treatment at this facility, who are subsequently referred back for follow-up at their centers of origin.

The EPIC was translated into Portuguese and validated according to the criteria described by Guillemin et al. [13]. These results were published in 2013 by Alves et al. [10]. To create the Portuguese version of the EPIC-CP, we used the same questions and language that had been previously validated in the EPIC. Consequently, language translation and linguistic validation were not addressed in this study. The EPIC-CP Portuguese version is show in Additional file 1 of this manuscript.

For the statistical analysis and validity data, we used Statistical Package for the Social Sciences for Windows/MAC (version 23.0 K, SPSS, Chicago, IL, USA). To confirm each domain’s reliability and internal consistency, we used Cronbach’s alpha coefficient using the data from the complete sample and the 6-month postoperative group. Furthermore, for the prospective data analysis of the three groups (baseline, 6 months, and 12 months), we applied the t-test and Wilcoxon’s test for non-parametric measures, and the Friedman’s test for comparisons among the three groups. The level of significance for the statistical tests was set at 5% or *p* < 0.05.

Finally, confirmatory factor analysis (CFA) was performed considering the structure already used [14]. For this, the complete database was used (n = 402), without the need for other procedures for the sample. The objective was to reinforce robustness and make it invariable, despite the specificities of each participating group and the time-point of filling the questionnaire. We set up the structure based on the five domains, and each domain had its items linked to it and related to the other domains. The expected values for CFA when the model was built in IBM SPSS AMOS were chi-square (χ^2^) *p* < 0.05, chi-square mean divided by its degrees of freedom (CMIN) < 5, root mean square error of approximation (RMSEA) ≤ 0.08, comparative fit index (CFI) between 0 and 1, goodness-of-fit index (GFI) ≥ 0.90, and adjusted GFI (AGFI) ≥ 0.90 [15].

## Results

The clinical characteristics of the patients included in this study are described in Table 1.

**Table 1:** Clinical characteristics of the patients studied

Variables	Studied group
N	402
Age (years)^a^	62.9 ± 8.5
PSA (ng/ml)^a^	7.0 ± 11.1
BMI^a^	27.3 ± 3.2
ASA (n,%)	
I	n = 89 (22.1%)
II	n = 306 (76.2%)
III/IV	n = 7 (1.7%)
ISUP (n, %)	
I	n = 11 (2.7%)
II	n = 153 (38.1%)
III	n = 139 (34.6%)
IV	n = 30 (7.5%)
V	n = 69 (17.1%)

^a^These variables are expressed are mean and standard deviation

The mean age of the preoperative patients was 62.9 years old (± 8.5), and the mean prostate specific antigen level was 7.0 ng/ml (± 11.1). Most of the patients (98.3%) showed American Society of Anesthesiologists’ levels I and II with controlled systemic disease. Most cases (72.7%) presented Gleason grade 7 (International Society of Urologic Pathologists grades II and III).

Cronbach’s alpha coefficient was used to analyze the internal consistency of the questionnaire. The reliability of this scale ranges from 0 to 1, and values > 0.7 are considered acceptable (ideally, reliability scores will be > 0.8). The internal consistency analysis based on data from the full sample for comparison purposes was as follows: urinary incontinence = 0.689 (four items), urinary irritation = 0.656 (three items), bowel = 0.640 (three items), sexual = 0.636 (three items), hormonal = 0.642 (three items), and total = 0.783 (16 items). The internal consistency analysis based on data from the 6-month postoperative group was performed according to the first validation of the EPIC-CP [1]. All domains had a reliability total score > 0.7, demonstrating an acceptable internal consistency (Table 2).

**Table 2:** Cronbach’s alpha analysis

EPIC-CP	Cronbach’s alpha			
	Median	SD	IC 95%	
Urinary				
Incontinence	1.1	1.8	0.82	0.77–0.86
Irritation/obstruction	0.3	1.3	0.75	0.67–0.81
Bowel	1.1	0.7	0.77	0.70–0.83
Sexual	5.8	3.0	0.76	0.68–0.82
Vitalily/hormonal	0.29	1.0	0.35	0.14–0.51
Total	10.1	5.8	0.77	0.71–0.82

**Fig. 1:**
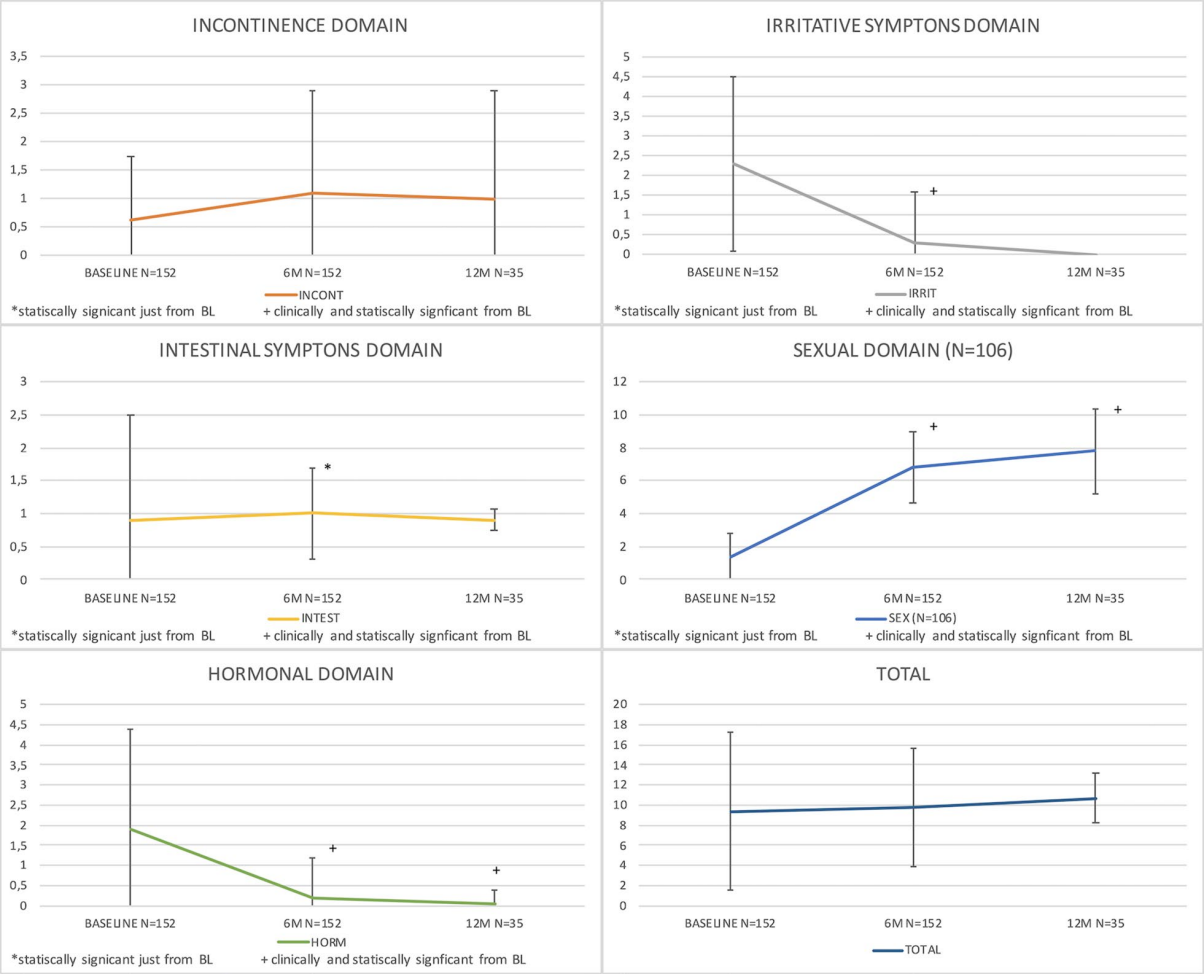
Main domains of the Expanded Prostate Cancer Index Composite for Clinical Practice (EPIC-CP)

Table 3 and Fig. 1 describe the domain evolution for the preoperative, 6-month postoperative, and 12-month postoperative groups. The domain related to urinary continence worsened, i.e., the score increased over the first 6 months, starting at 0.6 (± 1.1) in the preoperative period, increasing to 1.1 (± 1.8) at 6 months and 1.0 (± 1.9) at 12 months after the surgery. The only significant difference occurred between the preoperative period and 6 months postoperatively, but that variation did not have any clinical implications [16]. The other analyses for this domain did not present significant differences. The irritative symptoms improved (i.e., scores showed a statistically significant decrease) in all combined group analyses. The “intestinal” domain had a stable clinical baseline of 0.9 (± 1.6) that evolved to 1.0 (± 0.7) at 6 months and 0.9 (± 0.16) at 12 months postoperatively, although this difference was not clinically important according to the criteria described by Chipman [16]. In terms of the evolution of the “sexual” domain, we considered only the previously potent patients (question 8 with a score of 0), which included 106 patients out of 152 (approximately 70%). Their scores worsened gradually from 1.4 (± 1.4) in the preoperative period to 7.8 (± 2.6) at 12 months postoperatively. All analyses showed significant differences; however, the increase by 1 point between the results of 6 months and 12 months was not clinically significant [12]. Only when the three groups were evaluated together did the total final questionnaire score present a statistically significant difference.

**Table 3:** The domain evolution for the preoperative and 6-month and 12 months postoperative groups

Variables	Baseline (n = 152)	6 M (n = 152)	12 M (n = 35)	p values			
				Baseline vs. 6 M vs. 12 Ma	Baseline vs. 6 Mb	Baseline vs. 12 Mb	6 M vs. 12 M ^b^
Question 1	1.7 ± 1.2	1.4 ± 0.9	1.2 ± 0.8	0.054	< 0.01	0.162	0.058
Urinary Incontinence	0.63 ± 1.1	1.1 ± 1.8	1.0 ± 1.9	0.44	< 0.01	0.32	0.34
Urinary Irritation / Obstruction	2.3 ± 2.2	0.3 ± 1.3	0 ± 0	< 0.01	< 0.01^c^	< 0.01^c^	0.017
Bowel	0.9 ± 1.6	1.0 ± 0.7	0.9 ± 0.16	< 0.01	< 0.01	0.108	0.18
Sexual (N = 106)	1.4 ± 1.4	6.8 ± 2.2	7.8 ± 2.6	< 0.01	< 0.01^c^	< 0.01^c^	0.032
Vitalily/Hormonal	1.9 ± 2.5	0.2 ± 1.0	0.05 ± 0.33	< 0.01	< 0.01^c^	< 0.01^c^	0.048
Total	9.4 ± 7.8	9.8 ± 5.9	10.7 ± 2.5	0.03	0.162	0.07	0.86

The variables are show as median and standard deviation

^a^Friedman test

^b^Wilcoxcon test

^c^Clinical and statistical difference

Finally, the results of the CFA of the proposed structure presented robust results: chi-square 169.003; degrees of freedom: 94, *p* < 0.001 (expected < 0.05), CMIN: 1948 (expected < 5); GFI: 0.951 (expected ≥ 0.90); AGFI: 0.9293 (expected ≥ 0.90); and RMSEA: 0.045 (confidence interval 0.034–0.055; expected ≤ 0.08).

## Discussion

The postoperative administration of questionnaires aims to guide clinical decision making, clarify the problems and consequences the patient faces after surgery, and evaluate the patient’s evolution objectively [17]. These validated questionnaires, originally created in another language, include a process of language adaptation and application to ascertain whether the questionnaire can be reliably understood in the new language [13].

The EPIC-CP was adapted from previous questionnaires (EPIC 50 and EPIC 26), and it is a quick and practical tool that can be self-administered in a short time [1, 9, 11].

Prospective data analysis is essential for the global analysis of prostate cancer patients and their complications, mainly postoperatively. Physicians usually underestimate the symptoms of this disease, as was described in another study [18]. The aim of the EPIC-CP questionnaire is to give the surgeon a picture of the patient’s actual QoL.

Regarding the internal consistency data for this questionnaire validation, our results were satisfactory. Considering the whole sample dataset, the relatively low alpha values observed were expected due to the less number of items in each domain. Alpha results show less variation and are more accurate for approximately 10 items. Thus, as this questionnaire is a simplified version of the EPIC-CP, it is expected that the values would be lower for domains with only 3–4 items. On the other hand, considering the recommended 6-month follow-up group dataset, they were comparable to the validation results of the versions in other languages, such as Chinese and Spanish [14, 17], all of which showed acceptable rates for the “urinary” domain and optimal Cronbach’s alpha values, except for the “hormonal” domain. Our analysis was more reliable (higher Cronbach’s alpha values) than that of the Chinese study, and presented similar values as that of the Spanish study. Despite these findings, it is strongly suggested that the use of alpha values for the complete scale be considered more than acceptable.

In all studies, the “hormonal” domain showed the worst results, and our study was no exception. In the literature, Chang et al. stated that because this domain is based on hormonal blockade symptoms, it should show a uniform response; however, the perception and depressive symptoms vary according to the surgical results, which alters these responses in patients who have undergone surgery, and generates a rate of inconsistency [1].

The first American EPIC-CP validation presented similar findings, with a low consistency rate for the “hormonal/vitality” domain, and this was attributed to the systemic nature of prostate cancer and the wide variation among the patients.

The time-points analyzed in the prospective aspect of this study were short, but long enough to show the impact on the QoL over time. The EPIC-CP was created to be quickly and feasibly administered, and it was based on other well-established questionnaires (such as the ICIQ—International Consultation on Incontinence Questionnaire and IPSS—International Prostatic Symptom Score) [18]. This is the first prospective analysis of a Brazilian cohort using this instrument. One remarkable finding was that the “continence” domain showed no clinically significant difference from the baseline at 6 months postoperatively [16]. All other domains showed good results, as previously described in this manuscript.

Our study had certain limitations. First, we only validated the questionnaire with patients who had been treated for prostate cancer with robotic surgery, and not for other types of treatment. We believe that with the publication of this manuscript, it will be easier to expand the use of the EPIC-CP Portuguese version. Another important limitation was that a relative small proportion of those who started the prospective analysis completed the 12-month follow-up. These patients were referred for surgical treatment, and later referred back to their center of origin for clinical follow-up.

## Conclusions

The data presented revealed that the EPIC-CP in the Portuguese language is worth using preoperatively and in follow-up patients. The postoperative use details the progress of the organic function, mainly regarding incontinence and impotence, and the patient feelings regarding an eventual surgical problem. Thus, the handling and quality of medical care can be improved using this questionnaire.

## Supplementary information

**Additional file 1.** The EPIC-CP Portuguese version.

**Additional file 2.** The EPIC-CP original version.

## Data Availability

The datasets used and/or analyzed during the current study available from the corresponding author on reasonable request.

## Abbreviations

AGFI: Adjusted goodness-of-fit index
ASA: American Society of Anesthesiologists physical status classification system
CFA: Confirmatory factor analysis
CFI: Comparative fit index
CMIN: Chi-square mean divided by its degrees of freedom
EPIC: Expanded Prostate Cancer Composite Index
EPIC-CP: Expanded Prostate Cancer Index Composite for Clinical Practice
GFI: Goodness-of-fit index
HRQOL: Health-related quality of life
ICIQ: International Consultation on Incontinence Questionnaire
IPSS: International Prostate Symptom Score
ISUP: International Society of Urological Pathology (ISUP) grading of prostate cancer
QoL: Quality of life
RMSEA: Root mean square error of approximation
SD: Standard deviation
