# Supplementary material for: Portuguese version of the Expanded Prostate Cancer Index Composite for Clinical Practice (EPIC-CP): psychometric validation and prospective application for early functional outcomes at a single institution
Source: BMC Urol. 2020 Oct 20;20:163. doi: 10.1186/s12894-020-00734-y (PMC7574474; doi:10.1186/s12894-020-00734-y)
Supplement: Supplementary file 1 — Additional file 1. The EPIC-CP Portuguese version. [file 12894_2020_734_MOESM1_ESM.pdf]

Índice Composto Expandido de Câncer de Próstata para Prática Clínica (EPIC-PC)

"Expanded Prostate Cancer Index Composite dor Clinical Practice (EPIC-CP)" **Data___/___/___**

Instrumento clínico para medir questões relativas à Qualidade de Vida em pacientes com câncer de próstata.

Paciente: Favor responder as questões abaixo, sinalizando a opção mais apropriada. Todas as questões devem ser respondidas baseadas nos sintomas das **ÚLTIMAS QUATRO SEMANAS**. Selecionar apenas uma opção para cada pergunta.

1. No geral, você está tendo problemas para urinar?

☐ Não ☐ Muito pouco ☐ Pouco ☐ Moderadamente ☐ Muito

2. Qual das opções a seguir descreve melhor o seu controle urinário?

0☐ Controle total 1☐ Gotejamento ocasional 2☐ Gotejamento frequente 4☐ Nenhum controle ___

3. Quantos absorventes ou fraldas para adultos você normalmente tem usado devido a perda urinária?

0☐ Nenhum 1☐ Um por dia 2☐ Dois por dia 4☐ Três ou mais por dia ___

4. O gotejamento ou escape urinário está sendo problemático (incômodo) para você?

0☐ Nada 1☐ Muito pouco 2☐ Pouco 3☐ Moderado 4☐ Muito ___

*__O MÉDICO__ deverá somar as respostas das questões de 2-4 para calcular o ***Escore de Sintomas de Incontinência Urinária (máximo 12):*** _____

5. Quão problemático (incômodo) têm sido cada um dos itens descritos abaixo?

	Nada	Muito Pouco	Pouco	Moderadamente	Muito
a. Doeu ou ardeu para urinar________________________	0☐	1☐	2☐	3☐	4☐
b. Jato de urina fraco ou esvaziamento incompleto da bexiga	0☐	1☐	2☐	3☐	4☐
c. Necessidade frequente de urinar durante o dia_________	0☐	1☐	2☐	3☐	4☐

*__O MÉDICO__ deverá somar as respostas das questões de 5a-5c para calcular o ***Escore de Sintomas Urinários Irritativos/Obstrutivos (máximo 12):*** _____

6. Quão problemático (incômodo) têm sido cada um dos itens descritos abaixo?

	Nada	Muito Pouco	Pouco	Moderadamente	Muito
a. Dor retal ou urgência para evacuar ______________	0☐	1☐	2☐	3☐	4☐
b. Aumento da frequência das evacuações __________	0☐	1☐	2☐	3☐	4☐
c. Problemas com seus hábitos intestinai ___________	0☐	1☐	2☐	3☐	4☐

*__O MÉDICO__ deverá somar as respostas das questões de 6a-6c para calcular o ***Escore de Sintomas Intestinais (máximo 12):*** _____

7. Como você classificaria a sua capacidade de ter um orgasmo (clímax)?

0☐ Muito bom 1☐ Bom 2☐ Razoável 3☐ Deficiente 4☐ Muito deficiente a nula ___

8. Como você descreveria a QUALIDADE habitual de suas ereções?

0☐ Firme suficiente para relação sexual 1☐ Firme suficiente para masturbação e preliminares 2☐ Não o suficientemente firme para qualquer atividade sexual 4☐ Absolutamente nenhuma ___

9. Como um todo, quão problemática (incômoda) têm sido sua função sexual ou sua falta de função sexual?

0☐ Nada problemática 1☐ Muito pouco problemática 2☐ Pouco problemática 3☐ Moderadamente Problemática 4☐ Muito problemática ___

O MÉDICO** deverá somar as respostas das questões de 7-9 para calcular o ***Escore de Sintomas Sexuais (máximo 12): _____

10. Quão problemática (incômoda) foram para você cada uma das seguintes situações descritas abaixo?

	Nada	Muito pouco	Pouco	Moderadamente	Muito
a. Ondas de calor ou sensibilidade mamária/aumento	0☐	1☐	2☐	3☐	4☐
b. Sentindo-se deprimido/triste_______________	0☐	1☐	2☐	3☐	4☐
c. Falta de energia/desanimado_______________	0☐	1☐	2☐	3☐	4☐

O MÉDICO** deverá somar as respostas das questões de 10a-10c para calcular o ***Escore de Sintomas de Vitalidade/Hormonal(máximo 12): _____

O MÉDICO deverá somar os escores dos cinco domínios para calcular o ***Escore Geral de Qualidade de Vida (máximo 60)***:__________
