# Supplementary material for: Portuguese version of the Expanded Prostate Cancer Index Composite for Clinical Practice (EPIC-CP): psychometric validation and prospective application for early functional outcomes at a single institution
Source: BMC Urol. 2020 Oct 20;20:163. doi: 10.1186/s12894-020-00734-y (PMC7574474; doi:10.1186/s12894-020-00734-y)
Supplement: Supplementary file 2 — Additional file 2. The EPIC-CP original version. [file 12894_2020_734_MOESM2_ESM.pdf]

Expanded Prostate Cancer Index Composite for Clinical Practice (EPIC-CP)

A Clinical Tool to Measure Urinary, Bowel, Sexual and Vitality/Hormonal Health **Date:** ___/___/___

Patients: Please answer the following questions by checking the appropriate checkbox. All questions are about your health and symptoms in the **LAST FOUR WEEKS**. Select one answer for each question.

1. Overall, how much of a problem has your urinary function been for you?
☐ No problem ☐ Very small problem ☐ Small problem ☐ Moderate problem ☐ Big problem

2. Which of the following best describes your urinary control?
0☐ Total control 1☐ Occasional dribbling 2☐ Frequent dribbling 4☐ No urinary control ____

3. How many pads or adult diapers per day have you been using for urinary leakage?
0☐ None 1☐ One pad per day 2☐ Two pads per day 4☐ Three or more pads per day ____

4. How big a problem, if any, has urinary dripping or leakage been for you?
0☐ No problem 1☐ Very small problem 2☐ Small problem 3☐ Moderate problem 4☐ Big problem ____

*CLINICIANS: ADD the answers from questions **2-4** to calculate the **Urinary Incontinence Symptom Score (out of 12):*** ☐

**5. How big a problem, if any, has each of the following been for you?**	**No problem**	**Very small problem**	**Small problem**	**Moderate problem**	**Big problem**	
a. Pain or burning with urination	0☐	1☐	2☐	3☐	4☐	____
b. Weak urine stream/incomplete bladder emptying	0☐	1☐	2☐	3☐	4☐	____
c. Need to urinate frequently	0☐	1☐	2☐	3☐	4☐	____

*CLINICIANS: ADD the answers from questions **5a-5c** to calculate the **Urinary Irritation/Obstruction Symptom Score (out of 12):*** ☐

**6. How big a problem, if any, has each of the following been for you?**	**No problem**	**Very small problem**	**Small problem**	**Moderate problem**	**Big problem**	
a. Rectal pain or urgency of bowel movements	0☐	1☐	2☐	3☐	4☐	____
b. Increased frequency of your bowel movements	0☐	1☐	2☐	3☐	4☐	____
c. Overall problems with your bowel habits	0☐	1☐	2☐	3☐	4☐	____

*CLINICIANS: ADD the answers from questions **6a-6c** to calculate the **Bowel Symptom Score (out of 12):*** ☐

7. How would you rate your ability to reach orgasm (climax)?
0☐ Very good 1☐ Good 2☐ Fair 3☐ Poor 4☐ Very poor to none ____

8. How would you describe the usual quality of your erections?
0☐ Firm enough for intercourse 1☐ Firm enough for masturbation and foreplay only 2☐ Not firm enough for any sexual activity 4☐ None at all ____

9. Overall, how much of a problem has your sexual function or lack of sexual function been for you?
0☐ No problem 1☐ Very small problem 2☐ Small problem 3☐ Moderate problem 4☐ Big problem ____

*CLINICIANS: ADD the answers from questions **7-9** to calculate the **Sexual Symptom Score (out of 12):*** ☐

**10. How big a problem, if any, has each of the following been for you?**	**No problem**	**Very small problem**	**Small problem**	**Moderate problem**	**Big problem**	
a. Hot flashes or breast tenderness/enlargement	0☐	1☐	2☐	3☐	4☐	____
b. Feeling depressed	0☐	1☐	2☐	3☐	4☐	____
c. Lack of energy	0☐	1☐	2☐	3☐	4☐	____

*CLINICIANS: ADD the answers from questions **10a-10c** to calculate the **Vitality/Hormonal Symptom Score (out of 12):*** ☐

*CLINICIANS: Add the five domain summary scores to calculate the **Overall Prostate Cancer QOL Score (out of 60):*** ☐

A downloadable version of EPIC-CP can be found at http://www.bidmc.org/epic
